# Immune checkpoint inhibitors combined with VEGF/VEGFR-targeted therapy in microsatellite-stable advanced colorectal cancer: clinical advances and the potential for immune-sensitive tumor conversion

**DOI:** 10.3389/fimmu.2026.1962600

**Published:** 2026-10-22

**Authors:** Ruoxin Wang, Xiaofang Zhou, Meiqin Yuan, Zeng Wang

**Affiliations:** 1 School of Pharmaceutical Sciences, Wenzhou Medical University, Wenzhou, Zhejiang, China; 2 Department of Pharmacy, Zhejiang Cancer Hospital, Hangzhou Institute of Medicine (HIM), Chinese Academy of Sciences, Hangzhou, Zhejiang, China; 3 Department of Colorectal Medicine, Zhejiang Cancer Hospital, Hangzhou Institute of Medicine (HIM), Chinese Academy of Sciences, Hangzhou, Zhejiang, China; 4 Zhejiang Key Laboratory of Functional Nucleic Acids for Basic and Clinical Application of Sciences, Hangzhou, Zhejiang, China

**Keywords:** antiangiogenic therapy, immune checkpoint inhibitors, microsatellite-stable colorectal cancer, tumor microenvironment, VEGF, VEGFR

## Abstract

Colorectal cancer (CRC) remains a major cause of cancer-related morbidity and mortality worldwide, and mismatch repair-proficient/microsatellite-stable (pMMR/MSS) tumors account for the majority of metastatic CRC cases. In contrast to dMMR/MSI-H CRC, pMMR/MSS tumors are typically characterized by low immunogenicity, limited neoantigen presentation, insufficient effector T-cell infiltration, and an immunosuppressive tumor microenvironment, which collectively restrict the efficacy of immune checkpoint inhibitor (ICI) monotherapy. Vascular endothelial growth factor (VEGF)/VEGF receptor (VEGFR) signaling contributes not only to abnormal angiogenesis but also to immune suppression by impairing dendritic-cell maturation, reducing immune-cell trafficking, promoting T-cell dysfunction, and facilitating the accumulation of immunosuppressive cell populations. These mechanisms provide a strong rationale for combining VEGF/VEGFR-targeted therapy with ICIs to normalize tumor vasculature and remodel the immune microenvironment. Clinical evidence for this strategy in advanced pMMR/MSS CRC remains heterogeneous. Treatment activity varies across therapeutic settings, antiangiogenic agents, and combination regimens, while many studies are limited by small sample sizes, single-arm designs, and substantial differences in patient characteristics. Emerging data suggest that metastatic pattern, molecular features, baseline immune contexture, and treatment line may influence clinical activity; however, no biomarker has yet been validated for routine patient selection. Multi-agent strategies may provide broader immune modulation but also increase toxicity, treatment complexity, and cost. This review summarizes the biological rationale, clinical efficacy, safety, and potential predictive factors of VEGF/VEGFR-targeted therapy combined with ICIs in advanced pMMR/MSS CRC. Particular emphasis is placed on the heterogeneity of clinical outcomes and on distinguishing mechanistic immune remodeling from the potential enhancement of tumor immune sensitivity. Future studies should prioritize biomarker-guided patient selection, randomized comparative trials, and comprehensive assessment of efficacy, safety, quality of life, and long-term clinical benefit.

## 1 Introduction

According to the latest epidemiological data, colorectal cancer (CRC) is the third most commonly diagnosed cancer and the second leading cause of cancer death worldwide, accounting for approximately 9.4% of global cancer deaths (1). In China, CRC is the second most common cancer (2). Approximately 85% of patients are initially diagnosed with intermediate- or advanced-stage disease and are no longer eligible for surgery (3). Metastatic colorectal cancer is a major form of advanced CRC, with a 5-year relative survival rate of only 16.9% (4).

In recent years, cancer immunotherapy (CIT) has significantly transformed cancer treatment, and has shown positive effects in the treatment of various malignant tumors, such as melanoma, renal cell carcinoma, and non-small cell lung cancer (5). Existing clinical studies have shown that ICI treatment produces durable antitumor activity and significantly prolongs survival in mismatch repair deficient (dMMR)/microsatellite instability high (MSI-H) CRC (6, 7). However, immune checkpoint inhibitors have shown little efficacy in mismatch repair-proficient/microsatellite-stable (pMMR/MSS) CRC, which accounts for approximately 95% of CRC cases (8). pMMR/MSS CRC usually displays a low tumor mutational burden, limited neoantigen generation, an immune-desert tumor microenvironment, absent or inactive cytotoxic T lymphocytes, and enrichment of immunosuppressive stromal and myeloid components such as M2 macrophages and myeloid-derived suppressor cells (MDSCs). Together, these features contribute to ICI resistance (8, 9).

To improve responses to immune checkpoint inhibitors (ICIs) in patients with pMMR/MSS colorectal cancer, clinical studies have explored combining ICIs with agents that modulate the tumor microenvironment. Among these approaches, VEGF/VEGFR-targeted therapy has received particular attention. VEGF/VEGFR blockade can promote the normalization of abnormal tumor vessels, improving perfusion, reducing vascular permeability and alleviating hypoxia (10). These changes may make it easier for effector immune cells to enter the tumor. VEGF/VEGFR inhibition can also increase intratumoral CD8^+^ T-cell infiltration and effector function while reducing VEGF-mediated T-cell exhaustion (11). It may limit the accumulation of regulatory T cells (Tregs) and myeloid-derived suppressor cells (MDSCs), and shift tumor-associated macrophages (TAMs) from an immunosuppressive M2-like state toward M1-like (12, 13). Together, these vascular and immune changes may relieve some of the barriers to antitumor immunity in pMMR/MSS colorectal cancer, creating conditions more favorable for the activity of ICIs (**Figure 1**).

**Figure 1:**
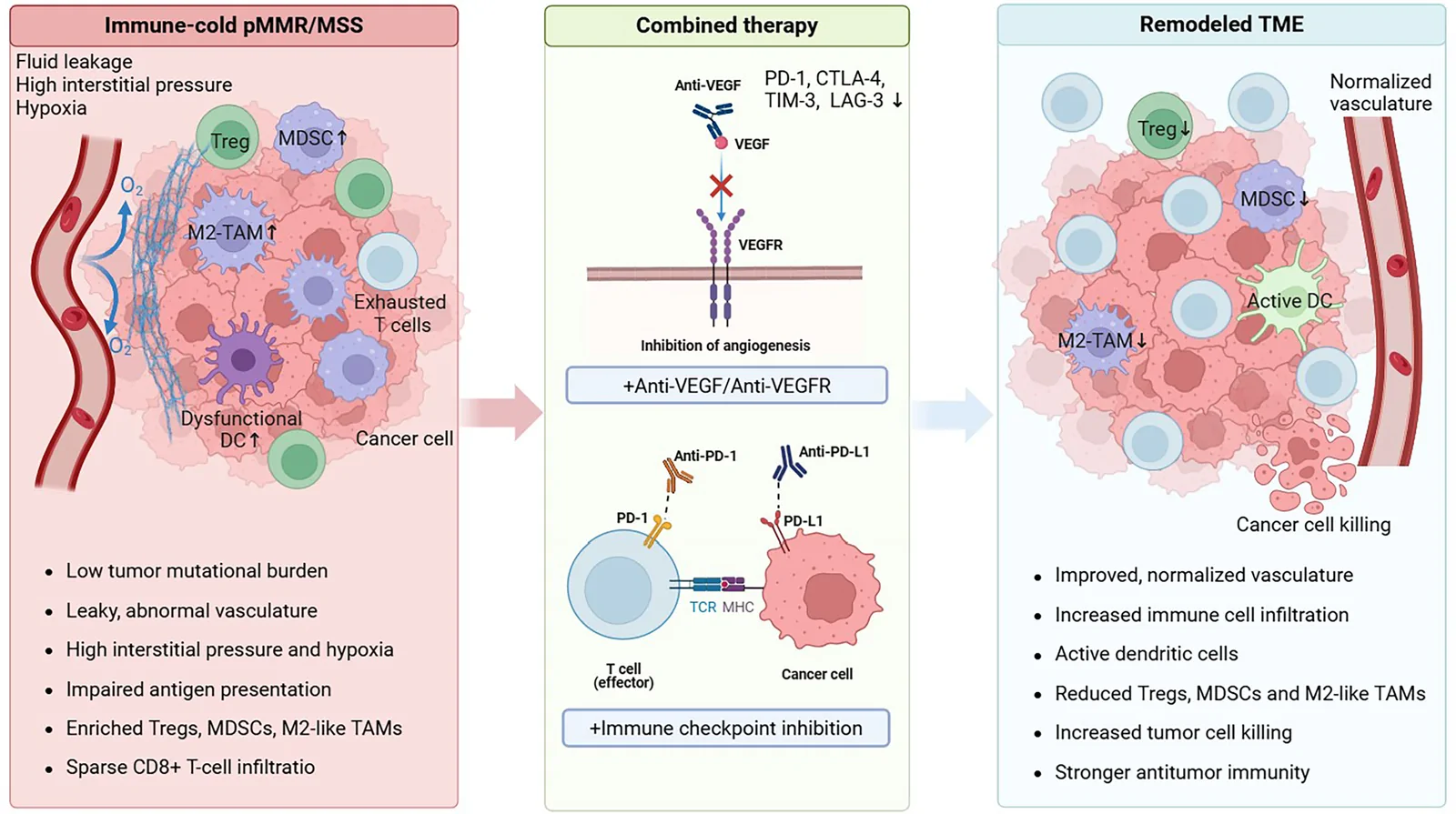
Schematic illustration of tumor immune microenvironment remodeling induced by combined VEGF/VEGFR blockade and immune checkpoint inhibition in pMMR/MSS colorectal cancer. Abnormal vasculature, hypoxia, impaired antigen presentation, and immunosuppressive cell infiltration contribute to the immune-cold phenotype of pMMR/MSS CRC. VEGF/VEGFR blockade may normalize tumor vasculature and facilitate immune-cell infiltration, while checkpoint inhibition restores T-cell activity, collectively promoting a more immunologically active tumor microenvironment.

This narrative review focuses on ICIs combined with VEGF/VEGFR-targeted therapy for advanced pMMR/MSS colorectal cancer. We searched PubMed and Embase, with the search cutoff date set to August 2026. Publications within the past 10 years were considered. Pre-defined inclusion criteria: clinical studies reporting efficacy or safety data for ICI plus VEGF/VEGFR-targeted therapy in advanced pMMR/MSS CRC, including full-text articles and complete conference abstracts with extractable endpoints. Exclusion criteria: studies without separable pMMR/MSS subgroup data, cohorts limited to dMMR/MSI-H CRC, non-CRC studies, duplicate publications, and records lacking evaluable clinical outcomes. Duplicate records were removed by cross-checking trial identifiers and publication information. For multiple reports from the same trial, the most recent complete publication was prioritized for data extraction.

## 2 Clinical trials progress on the combination of ICIs with VEGF/VEGFR inhibitors in advanced MSS colorectal cancer

### 2.1 Progress in clinical trials on doublet combination regimens

Patients with pMMR/MSS mCRC receiving third-line or later therapy generally have refractory disease, having experienced disease progression after standard first- and second-line therapies or being unable to tolerate these regimens. Following failure of fluoropyrimidine-, oxaliplatin-, and irinotecan-based chemotherapy, together with appropriate targeted therapies, subsequent treatment options become increasingly limited (14). Although the CSCO and ESMO guidelines recommend regorafenib and fruquintinib as treatment options for MSS mCRC in the third-line and later settings, their clinical benefits are mainly reflected in disease control and prolongation of progression-free survival, while objective response rates remain relatively modest (15–18). In recent years, some clinical studies have explored the combination of ICIs with multitargeted tyrosine kinase inhibitors(TKI), with the aim of enhancing antitumor activity in patients receiving third-line or later therapy. Meanwhile, a limited number of studies have moved these combination strategies into the first- or second-line setting to further evaluate their efficacy, safety, and potential target populations at earlier stages of treatment. **Table 1** provides a more comprehensive summary of studies of VEGF/VEGFR-targeted therapy combined with immune checkpoint blockade in pMMR/MSS mCRC.

**Table 1:** Clinical advances in pMMR/MSS dual-drug combination therapy.

Lines of therapy	NCT identifier (study name)	Study phase	Study design	Targets	KRAS/NRAS mutation	BRAF mutation	Liver metastasis	Sample size	Combination therapy	Main findings (95% CI)	Ref.
Third -line and later-line	NCT05425940 (STELLAR-303)	III	Prospective, multicenter, randomized, open-label	TKI (anti-VEGFR) + Anti-PD-L1	**Experimental arm:** 59% **Control arm:** 60%	**Experimental arm:** 3% **Control arm:** 4%	**Experimental arm:** 59% **Control arm:** 56%	**Experimental arm:** 451 **Control arm:** 450	**Experimental arm:** Zanzalintinib + Atezolizumab **Control arm:** Regorafenib	**Experimental arm:** mPFS: 3.7 months (3.4–3.8) mOS: 10.9 months (9.9–12.1) ORR: 4% (2–6%) **Control arm:** mPFS: 2.0 months (1.9–2.6) mOS: 9.4 months (8.5–10.2) ORR: 1% (0–3%)	2025 (30)
Third-line and later-line	NCT04776148 (LEAP-017)	III	Prospective, multicenter, randomized, controlled, open-label	TKI (anti-VEGFR) + Anti-PD-1	**Experimental arm:** 58% **Control arm:** 54%	**Experimental arm:** 2% **Control arm:** 5%	**Experimental arm:** 70% **Control arm:** 70%	**Experimental arm:** 241 **Control arm:** 239	**Experimental arm:** Lenvatinib + Pembrolizumab **Control arm:** Regorafenib or Trifluridine/Tipiracil	**Experimental arm:** mPFS: 3.8 months (3.7–5.1) mOS: 9.8 months (8.4–11.6) ORR: 10.4% (6.8–14.9%) **Control arm:** mPFS: 3.3 months (2.0–3.7) mOS: 9.3 months (8.2–10.9) ORR: 1.7% (0.5–4.2%)	2024 (29)
Third-line and later-line	NCT03797326 (LEAP-005)	II	Prospective, multicenter, open-label	TKI (anti-VEGFR) + Anti-PD-1	**Experimental arm:** NR **Control arm:** NR	**Experimental arm:** NR **Control arm:** NR	**Experimental arm:** NR **Control arm:** NR	**Experimental arm:** 105 **Control arm:** 35	**Experimental arm:** Lenvatinib + Pembrolizumab **Control arm:** Lenvatinib	**Experimental arm:** mPFS: 3.4 months (2.1–4.1) mOS: 8.7 months (7.0–10.0) ORR: 14% (8–23%) **Control arm:** mPFS: 3.4 months (2.1–4.2) mOS: 7.7 months (5.6–14.8) ORR: 7% (1–22%)	2026 (27)
Third-line and later-line	NCT04346381	II	Prospective, multicenter, basket cohort	TKI (anti-VEGFR) + Anti-PD-1	50%	2.3%	72.7%	44	Famitinib + Camrelizumab	mPFS: 3.3 months (2.1–4.1) mOS: 10.9 months (7.6–15.2) ORR: 13.6% (5.2–27.4%)	2025 (62)
Third-line and later-line	NCT04745130	II	Prospective, single-center, single-arm, open-label	TKI (anti-VEGFR) + Anti-PD-1	NR	NR	61.2%	103	Regorafenib + Sintilimab	mPFS: 4.1 months (3.4–4.8) mOS: 14.1 months (10.5–17.7) ORR: 21.4% (13.3–29.4%)	2025 (22)
Third-line and later-line	NCT03539822 (CAMILLA CRC cohort)	I/II (reported data: phase II CRC cohort)	Prospective, single-center, single-arm, open-label	TKI (anti-VEGFR) + Anti-PD-L1	59%	0%	79%	29 efficacy-evaluable	Cabozantinib + Durvalumab	mPFS: 3.7 months (3.4–5.7) mOS: 9.1 months (5.8–20.2) ORR: 27.6% (12.73–47.2%)	2024 (60)
Third-line and later-line	NCT03903705	Ib/II (reported data: phase II dose-expansion pMMR cohort)	Prospective, multicenter, open-label	TKI (anti-VEGFR) + Anti-PD-1	NR	NR	NR	25	Fruquintinib + Sintilimab	mPFS: 6.9 months (4.8–10.1) mOS: 20.0 months (8.1–NR) ORR: 20.0% (4.3–48.1%)	2023 (28)
Third-line and later-line	ChiCTR2000028965	II	Prospective, single-center, single-arm	TKI (anti-VEGFR) + Anti-PD-1	50.0% (8/16 tested)	0%	68.42%	19	Fruquintinib + Toripalimab	mPFS: 5.98 months (2.79–10.10) mOS: 11.10 months (7.66–not reached) ORR: 21.05% (6.05–45.57%)	2023 (63)
Third-line and later-line	NCT04126733	II	Prospective, multicenter, single-arm, open-label	TKI (anti-VEGFR) + Anti-PD-1	61%	4%	67%	70	Regorafenib + Nivolumab	mPFS: 1.8 months (1.8–2.4) mOS: 11.9 months (7.0–not reached) ORR: 7% (2.4–15.9%)	2023 (24)
Third-line and later-line	NCT03657641	I/II (reported data: pooled phase I + II; 10 + 63 patients)	Prospective, single-center, single-arm	TKI (anti-VEGFR) + Anti-PD-1	68%	5%	78%	73	Regorafenib + Pembrolizumab	mPFS: 2.0 months (1.8–3.5) mOS: 10.9 months (5.3–not reached) ORR: 0% (NR)	2022 (26)
Third-line and later-line	NCT03946917 (REGOTORI)	Ib/II (reported efficacy: pooled evaluable cohort)	Prospective, multicenter, single-arm, open-label	TKI (anti-VEGFR) + Anti-PD-1	54.5%	6.1%	69.7%	33 efficacy-evaluable	Regorafenib + Toripalimab	mPFS: 2.1 months (2.0–4.3) mOS: 15.5 months (10.3–not reached) ORR: 15.2% (5.7–32.7%)	2021 (14)
Third-line and later-line	NCT03475953 (REGOMUNE)	II	Prospective, multicenter, single-arm, open-label	TKI (anti-VEGFR) + Anti-PD-L1	64%	6%	74.5%	48	Regorafenib + Avelumab	mPFS: 3.6 months (1.8–5.4) mOS: 10.8 months (5.9–not reached) ORR: 0% (NR)	2021 (25)
Third-line and later-line	NCT03912857	II	Prospective, single-center, single-arm, open-label	TKI (anti-VEGFR) + Anti-PD-1	70%	10%	60%	10	Camrelizumab (SHR-1210) + Apatinib	mPFS: 1.83 months (1.80–1.86) mOS: 7.80 months (0–17.07) ORR: 0% (NR)	2020 (64)
Later-line	NCT05460767	I; dose-escalation/expansion results	Prospective, dose-escalation/expansion	PD-1/IL-2α-bias fusion protein + Anti-VEGF	41.1%	NR	54.8%	73	IBI363 + Bevacizumab	mPFS: NR mOS: NR ORR: 23.5% (14.1–35.4%)	2025 (65)
Third-line and later-line	NCT03475251	I (reported data: part 2a combination cohort)	Prospective, multicenter, dose-escalation	TKI (anti-VEGFR) + Anti-PD-1	50%	7.1%	NR	14	Regorafenib + Nofazinlimab	mPFS: 1.8 months (1.7–3.4) mOS: NR ORR: 0% (NR)	2023 (66)
Third-line and later-line	NCT03170960 (COSMIC-021 CRC cohort)	Ib	Prospective, multicenter, expansion cohort	TKI (anti-VEGFR) + Anti-PD-L1	61.3%	NR	NR	31	Cabozantinib + Atezolizumab	mPFS: 3.0 months (2.7–5.4) mOS: 14.0 months (5.5–16.7) ORR: 9.7% (2.0–25.8%)	2022 (21)
Third-line and later-line	NCT03712943	I/Ib (reported data: pooled dose-escalation + expansion)	Prospective, single-center, dose-escalation/expansion	TKI (anti-VEGFR) + Anti-PD-1	71.2%	NR	73.1%	52	Regorafenib + Nivolumab	mPFS: 4.3 months (NR) mOS: 11.1 months (NR) ORR: 10% (NR)	2022 (20)
Third-line and later-line	NCT03406871 (REGONIVO/EPOC1603)	Ib	Prospective, multicenter, dose-escalation/expansion	TKI (anti-VEGFR) + Anti-PD-1	24%	NR	52%	25 (pMMR/MSS: 24)	Regorafenib + Nivolumab	pMMR/MSS subgroup: mPFS: NR mOS: NR ORR: 33% (15.6–55.3%)	2020 (19)
Third-line and later-line	He et al.	/	Retrospective, single-center	TKI (anti-VEGFR) + Anti-PD-1	48%	NR	68.8%	77	Fruquintinib + PD-1 inhibitors	mPFS: 5.1 months (3.6–6.7) mOS: 14.6 months (9.6–15.6) ORR: 5.2% (NR)	2024 (67)
Third-line and later-line	Li et al.	/	Retrospective, single-center	TKI (anti-VEGFR) + Anti-PD-1	NR	NR	NR	43(pMMR/MSS:24)	Apatinib + PD-1 inhibitors	pMMR/MSS subgroup: mPFS: 5.0 months (3.85-6.15) mOS: NR ORR: NR	2023 (68)
Third-line and later-line	Wang et al.	/	Retrospective, single-center	TKI (anti-VEGFR) + Anti-PD-1	NR	NR	77.8%	18	Regorafenib + Nivolumab/Pembrolizumab	mPFS: 2.0 months (NR) mOS: NR ORR: 0% (NR)	2020 (69)
Second-line and later-line	NCT02982694	II	Prospective, multicenter, single-arm, open-label	Anti-VEGF + Anti-PD-L1	NR	NR	38.1%	21	Atezolizumab + Bevacizumab	mPFS: 4.0 months (NR) mOS: NR ORR: 9.5% (1.2–30.4%)	2025 (33)
First-line	NCT04271813 (APICAL-CRC)	II	Prospective, single-center, open-label	TKI (anti-VEGFR) + Anti-PD-1	50%	3.3%	63.3%	30	Anlotinib + Sintilimab	mPFS: 8.6 months (4.8–11.0) mOS: 22.9 months (13.5–36.3) ORR: 48.3% (29.4–67.5%)	2025 (34)

NR, not reported; NE, not estimable; NA, not applicable; mPFS, median progression-free survival; mOS, median overall survival; ORR, overall response rate; MSS, microsatellite stable; MSI, microsatellite instability.

*Bold values represent primary efficacy endpoints of the corresponding clinical trials, including ORR, mPFS and mOS.

#### 2.1.1 Third- and further-line therapy

Early-phase studies provided initial evidence that VEGF/VEGFR-targeted agents could be combined with immune checkpoint blockade in pMMR/MSS mCRC. In the phase Ib REGONIVO study, regorafenib plus nivolumab achieved an objective response rate (ORR) of 33%(95% CI, 15.6–55.3%) and a median progression-free survival (PFS) of 7.9 months in the MSS mCRC cohort, achieving a good antitumor activity (19). Another phase I/Ib study of regorafenib plus nivolumab reported an ORR of 10%(95% CI, no reported), mPFS of 4.3 months (20). This combination demonstrated only modest antitumor activity in metastatic pMMR colorectal cancer, but more pronounced responses were observed in patients without liver metastases, with lung metastases, and with lower levels of regulatory T-cell (Treg) infiltration in the tumor microenvironment (20). In the phase Ib COSMIC-021, cabozantinib plus atezolizumab demonstrated encouraging antitumor activity with manageable toxicity in patients with previously treated advanced non-MSI-H/MMR-proficient CRC, achieving an ORR of 9.7% (95% CI, 2.0–25.8), a median PFS of 3.0 months (95% CI, 2.7–5.4), and a median OS of 14.0 months (95% CI, 5.5–16.7) (21). Building on these early signals of antitumor activity and acceptable safety, several phase II studies were subsequently conducted to further evaluate the efficacy of ICI plus VEGF/VEGFR-targeted combinations.

In a single-arm phase II study, 103 patients with MSS mCRC who had progressed after at least two lines of standard systemic therapy received regorafenib plus sintilimab. The ORR was 21.4% (95% CI, 13.3–29.4%), with a median PFS of 4.1 months (95% CI, 3.4–4.8) and a median OS of 14.1 months (95% CI, 10.5–17.7) (22). The combination showed greater antitumor activity than that historically reported with either agent alone (22). Exploratory subgroup analyses further suggested longer survival and delayed disease progression in patients with RAS/RAF wild-type tumors and in those without liver metastases (22). These subgroup differences may partly reflect distinct immune microenvironments, as oncogenic RAS signaling and liver metastases have both been associated with reduced effector T-cell activity and enhanced myeloid-mediated immunosuppression, potentially weakening the immune-remodeling effects of antiangiogenic therapy (23). Larger prospective studies are warranted to validate these findings and further define the clinical value of this combination (22).

Other regorafenib-based combinations showed less activity. In a multicenter phase II study, regorafenib plus nivolumab achieved an ORR of 7% (95% CI, 2.4–15.9%), with a median PFS of 1.8 months and a median OS of 11.9 months (24). Although the study did not meet its predefined primary efficacy endpoint, several exploratory findings suggested potential benefit in treatment (24). Five confirmed partial responses were observed among the 23 patients without liver metastases (22%), whereas no responses occurred among the 47 patients with liver metastases (24). This pattern is biologically plausible because liver metastases can induce systemic immune suppression through macrophage-mediated elimination of activated CD8^+^ T cells, thereby reducing the pool of effector T cells available for immune-checkpoint blockade. In addition, patients with KRAS wild-type tumors showed a numerically higher proportion of partial responses or stable disease lasting at least 16 weeks than those with KRAS-mutant tumors (24). KRAS mutations may contribute to an immunosuppressive microenvironment through reduced cytotoxic T-cell infiltration and enhanced recruitment of suppressive myeloid populations, providing a possible mechanistic explanation for the numerically lower activity observed in KRAS-mutant tumors. However, both liver-metastasis and KRAS-status findings were exploratory and should not be interpreted as established predictive biomarkers. These observations warrant further prospective validation to determine whether they can be used to enrich for patients more likely to benefit from regorafenib plus nivolumab.

In the REGOMUNE study, regorafenib plus avelumab produced no objective responses, with a 6-month ORR of 0%(95% CI, no reported) (25). Nevertheless, the median PFS and median OS were 3.6 and 10.8 months, respectively, which were numerically longer than the median PFS of 1.9 months and median OS of 6.4 months reported with regorafenib monotherapy in the pivotal study (15, 25).Moreover, it showed that a high baseline abundance of tumor-infiltrating M2 macrophages was significantly associated with poorer outcomes(progression-free survival: 1.8 vs 3.7 months, p=0.002; overall survival: 3.7 months versus not reached, p=0.002) (25).

A phase II multicenter study evaluated regorafenib plus pembrolizumab in a diverse population of patients with advanced MSS colorectal cancer. The study did not meet its primary endpoint (ORR 0%(95% CI, no reported)), although the median OS of 10.9 months was considered noteworthy (26). Ongoing biomarker analyses aim to identify patient subgroups with a longer duration of benefit from this combination.

Beyond regorafenib-based combinations, the phase II LEAP-005 study evaluated lenvatinib plus pembrolizumab in patients with advanced CRC after two prior lines of systemic therapy (27). Among 105 patients who received the combination, the ORR was 14% (95% CI, 8–23%), median PFS was 3.4 months (95% CI, 2.1–4.1), and median OS was 8.7 months (95% CI, 7.0–10.0) (27). The regimen produced objective responses, although its overall activity was modest in this previously treated cohort.

In the phase Ib/II study, fruquintinib plus sintilimab showed a manageable safety profile and promising antitumor activity (the ORR was 20.0% (95% CI, 4.3-48.1%)) (28). Durable tumor responses were observed in previously treated mCRC, including in patients with pMMR tumors (with a median PFS of 6.9 months (95% CI, 4.8-10.1) and a median OS of 20.0 months (95% CI, 8.1-NR)) (28).

Although LEAP-017 was a negative phase III trial because lenvatinib plus pembrolizumab failed to meet the prespecified statistical significance threshold for the primary OS endpoint, several other clinically relevant endpoints showed numerical improvements (29). The ORR was 10.4% (95% CI, 6.8–14.9%) with lenvatinib plus pembrolizumab versus 1.7% (95% CI, 0.5–4.2%) with regorafenib or trifluridine/tipiracil, and the median duration of response was 11.1 versus 7.6 months, respectively (29). In contrast, the phase III STELLAR-303 trial enrolled 901 patients and demonstrated a statistically significant OS benefit with zanzalintinib plus atezolizumab compared with regorafenib, with a median OS of 10.9 months (95% CI, 9.9–12.1) versus 9.4 months (95% CI, 8.5–10.2), respectively (HR, 0.80; 95% CI, 0.69–0.93; P = 0.0045) (30). However, the objective response rates remained low (4% vs 1%(95% CI, 2–6% vs 0–3%)), suggesting that the overall magnitude of tumor shrinkage was limited and that the clinical benefit of this combination remains modest in pMMR/MSS population (30). In the ITT population, zanzalintinib plus atezolizumab was associated with better overall survival than regorafenib across different subgroups, regardless of geographic region, RAS status, liver metastases, or prior anti-VEGF antibody treatment (30).

The divergent outcomes of LEAP-017 and STELLAR-303 may partly reflect differences in the kinase-inhibition profiles of lenvatinib and zanzalintinib. In addition to VEGFRs, zanzalintinib inhibits MET and TAM family kinases, which are implicated in tumor immune evasion and myeloid-mediated immunosuppression (31). TAM signaling can promote an immunosuppressive macrophage phenotype and efferocytosis, thereby attenuating proinflammatory immune responses (32). Thus, inhibition of MET and TAM kinases may provide broader modulation of the tumor immune microenvironment and potentially enhance responsiveness to immune checkpoint blockade. However, because these findings were derived from separate trials involving different agents, patient populations, and study designs, cross-trial comparisons cannot establish that the broader kinase-inhibition profile of zanzalintinib directly accounted for the positive outcome of STELLAR-303.

#### 2.1.2 First- and second-line therapy

A phase II study evaluating atezolizumab plus bevacizumab in MSI-like mCRC showed limited activity in the conventionally defined MSS subgroup, with an ORR of only 9.5%(95% CI,1.2–30.4%) and a median PFS of 4.0 months (33). Notably, responses in MSS disease were predominantly observed in patients without liver metastases (ORR 15.4%), suggesting that metastatic pattern may warrant further investigation as a potential stratification factor (33).

In the first prospective trial (APICAL-CRC) to evaluate an immunotherapy–antiangiogenic combination as first-line treatment for metastatic colorectal cancer, Sintilimab plus anlotinib demonstrated promising antitumor activity in treatment-naive patients, achieving an ORR of 48.3%(95% CI, 29.4–67.5%) and a median PFS of 8.6 months (34). Moreover, despite the relatively high proportion of patients with an ECOG performance status of 2 (50.0%), grade≥3 adverse events occurred in only 13.3% of patients, suggesting a generally manageable safety profile (34). In addition, the presence of liver metastases was identified as an independent adverse prognostic factor for both PFS (HR = 5.66, 95% CI, 1.58–20.2) and OS (HR = 7.85, 95% CI, 1.38–44.8) (34). Notably, patients without hepatic metastasis respond well and benefit more from the combination of sintilimab plus anlotinib, suggesting that they are good candidates for this regimen (34).

#### 2.1.3 Safety

Safety profiles of dual-drug regimens were generally consistent with the known toxicities of VEGF/VEGFR-targeted agents and immune checkpoint inhibitors. Across studies, the most frequently reported adverse events included hypertension, proteinuria, palmar-plantar erythrodysesthesia, diarrhea, fatigue, hepatic dysfunction, and rash. Grade ≥3 toxicities were common in several TKI-based combinations, although their incidence varied substantially across studies because of differences in agents, dosing schedules, patient populations, and safety definitions. VEGF/VEGFR-related toxicities, including hypertension, proteinuria, and hand-foot skin reaction, represented the predominant treatment burden, while immune-related events such as rash, hypothyroidism, and hepatic abnormalities were also observed. (**Table 2**) Overall, these findings indicate that dual ICI–VEGF/VEGFR regimens are clinically feasible but require careful monitoring and proactive management of overlapping toxicities, particularly in heavily pretreated patients.

**Table 2:** Safety profiles of dual-drug regimens combining immune checkpoint inhibitors with VEGF/VEGFR-targeted therapy.

Targets	Clinical trial	Regimen	Safety population	Grade≥3 AEs, n (%)	common AEs
TKI(Anti-VEGFR)+ PD-1	NCT04776148 (29) (LEAP-017)	Lenvatinib + Pembrolizumab	238	183 (77%)	hypertension, proteinuria, diarrhea
	NCT03912857 (64)	Camrelizumab + Apatinib	10	9 (90%)	Hypertension; proteinuria; transaminase elevation
	NCT03797326 (27) (LEAP-005)	Lenvatinib + Pembrolizumab	105	19 (63%)	Hypertension, Diarrhea, Hypothyroidism
	NCT04346381 (62)	Famitinib + Camrelizumab	44	29 (65.9%)	Decreased platelet count, proteinuria, hypertension
	NCT03903705 (28)	Fruquintinib + Sintilimab	44	21 (47.7%)	Hypertension, palmar-plantar erythrodysesthesia, proteinuria
	ChiCTR2000028965 (63)	Fruquintinib + Toripalimab	19	7 (36.84%)	Fatigue, hepatic dysfunction, hypertension
	NCT04126733 (24)	Regorafenib + Nivolumab	70	32 (46%),	Fatigue, palmar-plantar erythrodysesthesia, maculopapular rash
	NCT04745130 (22)	Regorafenib + Sintilimab	103	9 (8.7%)	Asthenia, palmar-plantar erythrodysesthesia, gastrointestinal symptoms
	NCT03657641 (26)	Regorafenib + Pembrolizumab	73	NR	rash, hand-foot syndrome, Hypertension
	NCT03946917 (14) (REGOTORI)	Regorafenib + Toripalimab	42	80-mg cohort: 15 (38.5%) 120-mg cohort: 3 (100%)	Palmar-plantar erythrodysesthesia, rash, fever
TKI(Anti-VEGFR)+ PD-1	NCT03406871 (19) (REGONIVO)	Regorafenib + Nivolumab	25	NR	Palmar-plantar erythrodysesthesia, hypertension, rash
	NCT03712943 (20)	Regorafenib + Nivolumab	52	NR	Rash, hypertension, anemia
	NCT03475251 (66)	Regorafenib + Nofazinlimab	14	12 (85.7%)	Palmar-plantar erythrodysesthesia, arthralgia, pyrexia
	Li et al., 2023 (68)	Apatinib + PD-1 inhibitors	43	NR	Fatigue, hypertension, diarrhea
	He et al., 2024 (67)	Fruquintinib + PD-1 inhibitor	77	NR	Abdominal pain, rash, edema
	Wang et al., 2020 (69)	Regorafenib + Nivolumab/Pembrolizumab	18	0	Platelet decrease, Skin rash, Anemia, ALP increase
	NCT04271813 (34) (APICAL-CRC)	Anlotinib + Sintilimab	30	4 (13.3%)	Diarrhea, hypothyroidism, hypertension
TKI(Anti-VEGFR)+ PD-L1	NCT05425940 (30) (STELLAR-303)	Zanzalintinib + Atezolizumab	446	268 (60%)	diarrhea, hypertension, fatigue
	NCT03539822 (60) (CAMILLA)	Cabozantinib + Durvalumab	31	12 (39%)	Fatigue, transaminase elevation, nausea
	NCT03475953 (25) (REGOMUNE)	Regorafenib + Avelumab	48	NR	Fatigue, anorexia, palmar-plantar erythrodysesthesia
	NCT03170960 (21) (COSMIC-021)	Cabozantinib + Atezolizumab	31	16 (52%)	diarrhea, fatigue, nausea
PD-1/IL-2α-bias fusion protein + Anti-VEGF	NCT05460767 (65)	IBI363 (PD-1/IL-2α-bias bispecific) + Bevacizumab	73	22 (30.1%)	Arthralgia, anemia, rash
Anti-VEGF antibody + PD-L1	NCT02982694 (33)	Atezolizumab + Bevacizumab	45	22 (48.9%)	Fatigue, hypertension, abdominal pain

NR, not reported.

### 2.2 Progress in clinical trials of multi-agent regimens based on ICI plus VEGF/VEGFR inhibition

Given the limited efficacy of immune checkpoint inhibitor monotherapy and the unsatisfactorily short duration of tumor response achieved by dual blockade with immune checkpoint inhibitors plus VEGF/VEGFR-targeted agents, researchers have attempted to introduce a third agent with complementary mechanisms of action. This triple-combination strategy simultaneously targets tumor cells, abnormal angiogenesis, and the immunosuppressive tumor microenvironment, aiming to improve both the depth and durability of antitumor responses. **Table 3** summarizes the available clinical evidence of relevant combination regimens.

**Table 3:** Clinical advances in pMMR/MSS three or more agents combination therapy.

Lines of therapy	NCT identifier (study name)	Study phase	Study design	Targets	RAS mutation	BRAF mutation	Liver metastasis	Sample size	Combination therapy	Main findings (95% CI)	Ref.
Third-line and later-line	NCT03475004	II	Single-center, phase II; randomized run-in cohorts	Anti-PD-1 + MEK inhibitor + Anti-VEGF	49%	0%	76%	50	Pembrolizumab + Binimetinib + Bevacizumab	mPFS:5.9 months (4.2–8.7) mOS:9.3 months (6.7–12.2) ORR:12.0% (4.5–24.3%)	2024 (51)
Third-line and later-line	NCT04724239 (CAPability-01)	II	Prospective, multicenter, randomized	Anti-PD-1+HDAC inhibitor +Anti-VEGF	**Experimental arm:** 44.0%; **Control arm:** 39.1%	**Experimental arm:** 0%; **Control arm:** 4.3%	**Experimental arm:** 56.0%; **Control arm:** 52.2%	**Experimental arm:** 25 **Control arm:**23	**Experimental arm:** Chidamide + Sintilimab + Bevacizumab **Control arm:** Chidamide + Sintilimab	**Experimental arm:** mPFS:7.3 months (NR) mOS: NR ORR:44% (24.4-65.1%) **Control arm:** mPFS:1.5 months (NR) mOS: NR ORR:13% (2.8-33.6%)	2024 (48)
Third-line and later-line	NCT02873195 (BACCI)	II	Prospective, multicenter, randomized, double-blind	Chemotherapy+Anti-VEGF+Anti-PD-L1	**Experimental arm:** 61%; **Control arm:** 52.2%	7.1%	**Experimental arm:** 84.1% **Control arm:** 78.3%	**Experimental arm:** 82(pMMR/MSS:69) **Control arm:**46(pMMR/MSS:41)	**Experimental arm:** Capecitabine + Bevacizumab + Atezolizumab **Control arm:** Capecitabine + Bevacizumab + Placebo	pMMR/MSS subgroup: **Experimental arm:** mPFS:5.3 months (NR) mOS: not available ORR:10.1% (4.2–19.8%) **Control arm:** mPFS:3.3 months (NR) mOS: not available ORR:4.9% (0.6–16.5%)	2022 (38)
Third-line and later-line	NCT04362839 (RIN)	I	Prospective, single-center, nonrandomized	CTLA-4+Anti-VEGFR+Anti-PD-1	69.0%	10.3%	24.1%	39 total; 29 in RP2D efficacy cohort	Regorafenib + Ipilimumab + Nivolumab	RP2D cohort: mPFS:4.0 months (3.0-9.0) mOS:20.0 months (9.0-not reached) ORR:27.6% (NR)	2023 (40)
Third-line and later-line	ChiCTR2100046768 (RENMIN-215)	II	Prospective, single-center, single-arm, open-label	TKI (anti-VEGFR) + Anti-PD-1 + FMT	35%	0%	70%	20	Fruquintinib + Tislelizumab + FMT	mPFS: 9.6 months (4.1–15.1) mOS: 13.7 months (9.3–17.7) ORR: 20% (5.7–43.7%)	2023 (59, 70)
Third-line and later-line	NCT03555149 (MORPHEUS-CRC)	Ib	Prospective, multicenter, randomized, open-label platform	β-glucan immunomodulator + Anti-VEGF + Anti-PD-L1	**Experimental arm:** 60.0%; **Control arm:** 30.8%	NR	**Experimental arm:** 86.7%; **Control arm:** 69.2%	**Experimental arm:** 15 **Control arm:**13	**Experimental arm:** Atezolizumab + ImprimePGG + Bevacizumab **Control arm:** Regorafenib	**Experimental arm:** mPFS:1.5 months (1.4-2.8) mOS:5.7 months (4.4-10.5) ORR:0% (0-21.8%) **Control arm:** mPFS:2.8 months (1.6-3.1) mOS:10.2 months (4.8- not reached) ORR:0% (0-24.7%)	2021 (58)
Second-line or later	NCT03396926	II	Prospective, single-arm	Chemotherapy + Anti-VEGF + Anti-PD-1	69%	7%	80%	44 enrolled; 40 evaluable	Pembrolizumab + Capecitabine + Bevacizumab	mPFS:4.1 months (NR) mOS:10.1 months (NR) ORR:5.0% (NR)	2026 (71)
Second- or third-line	NCT05627635 (FOLFOX-3B)	Phase I/II; reported data from Phase I	Prospective, single-center	Chemotherapy+CTLA-4+Anti-VEGF+Anti-PD-1	35.7%	NR	64.3%	14	FOLFOX+Bevacizumab+Botensilimab+Balstilimab	mPFS:8.0 months (NR) mOS: NR ORR:71.4% (NR)	2025 (72)
Second-line	Gao et al.	/	Retrospective, multicenter	Chemotherapy+Anti-VEGF+Anti-PD-1	69.0%	NR	65.5%	29	Chemotherapy + Bevacizumab + anti-PD-1	mPFS:7.8 months (NR) mOS:28.8 months (NR) ORR:17.2% (NR)	2025 (73)
First-line	NCT04072198 (NIVACOR)	II	Prospective, multicenter, single-arm	Chemotherapy+Anti-VEGF+Anti-PD-1	84.9%	15.1%	56.2%	73 (pMMR/MSS:63)	FOLFOXIRI + Nivolumab + Bevacizumab	pMMR/MSS subgroup: mPFS:9.5 months (8.2-12.2) mOS: NR ORR:77.8% (65.5-87.3%)	2026 (37)
First-line	NCT05839470 (SYLT-026)	II	Prospective, multicenter, single-arm	Chemotherapy + Anti-VEGF + PD-1/CTLA-4 bispecific	35%		50%	20 enrolled; 15 initially evaluable	Cadonilimab + FOLFOXIRI/FOLFIRINOX + Bevacizumab	mPFS:17.2 months (13.6-20.7) mOS: NR ORR:100% among 15 evaluable.	2025 (42, 43)
First-line	NCT06206096	II	prospective, open-label, single-arm	Chemotherapy+Anti-VEGF+Anti-PD-1	100%	NR	72.7%	33	Sintilimab + Bevacizumab + CAPOX	mPFS:12.9 months (7.5–18.3) mOS: not reached ORR: 72.7% (55.8-84.9%)	2025 (74)
First-line	NCT03414983 (CheckMate 9X8)	II	Prospective, multicenter, randomized, open-label	Chemotherapy + Anti-VEGF + Anti-PD-1	**Experimental arm:**43% **Control arm:** 53%	NR	**Experimental arm:** 68% **Control arm:** 65%	**Experimental arm:** 127 (pMMR/MSS:121) **Control arm:** 68(pMMR/MSS:61)	**Experimental arm:** Nivolumab + mFOLFOX6 + Bevacizumab + SOC **Control arm:** SOC	pMMR/MSS subgroup: **Experimental arm:** mPFS:11.8 months(NR) mOS : NR ORR : NR **Control arm:** mPFS: 11.9 months (NR) mOS : NR ORR : NR	2024 (35)
First-line	NCT03721653 (AtezoTRIBE)	II	Prospective, multicenter, randomized, open-label	Chemotherapy+Anti-VEGF+Anti-PD-L1	**Experimental arm:**74% **Control arm:** 71%	**Experimental arm:**8% **Control arm:** 14%	NR	**Experimental arm:** 145 (pMMR/MSS:134) **Control arm:** 73 (pMMR/MSS:68)	**Experimental arm:** FOLFOXIRI + Bevacizumab + Atezolizumab **Control arm:** FOLFOXIRI + Bevacizumab	pMMR/MSS subgroup: **Experimental arm:** mPFS:12.9 months (80% CI 11.9-13.3) mOS:30.8 months (NR) ORR: NR **Control arm:** mPFS:11.4 months (80% CI 10.0-12.6) mOS:29.2 months (NR) ORR: NR	2024 (36)
First-line	NCT04068610 (COLUMBIA-1)	Phase Ib/II; reported efficacy from Phase II (Part 2)	Prospective, multicenter, randomized, open-label	Chemotherapy +Anti-VEGF+Anti-PD-L1+ Anti-CD73	NR	NR	**Experimental arm:** 96.2%; **Control arm:** 84.6%	**Experimental arm:** 26 **Control arm:** 26	**Experimental arm:** FOLFOX + Bevacizumab + Durvalumab + Oleclumab **Control arm:** FOLFOX + Bevacizumab	**Experimental arm:** mPFS:10.9 months (6.9-15.1) mOS:22.4 months (10.6- not reached) ORR:61.5% (40.6-79.8%) **Control arm:** mPFS:11.1 months (7.3-16.2) mOS: NR (20.6- not reached) ORR:46.2% (26.6-66.6%)	2024 (52)
First-line	NCT04547166 (ASTRUM-015)	Phase II/III; reported data from Phase II part	Prospective, multicenter, randomized, double-blind	Chemotherapy + Anti-VEGF + Anti-PD-1	**Experimental arm:** 54.4% **Control arm:** 63.1%;	**Experimental arm:** 1.8% **Control arm:** 3.5%;	**Experimental arm:**66.7% **Control arm:** 66.7%	**Experimental arm:** 57(pMMR/MSS:40) **Control arm:** 57(pMMR/MSS:50)	**Experimental arm:** Serplulimab + HLX04 + XELOX **Control arm:** Placebo + Bevacizumab + XELOX	pMMR/MSS subgroup: **Experimental arm:** mPFS:17.2 months (9.8-not evaluable) mOS: not reached ORR : NR **Control arm:** mPFS:10.1 months (8.1-16.6) mOS: not reached ORR : NR	2024 (75)
First-line	NCT04194359 (BBCAPX)	II	Prospective, multicenter, open-label, single-arm	Chemotherapy+Anti-VEGF+Anti-PD-1	84%	NR	60%	25	Sintilimab + Bevacizumab + CapeOx	mPFS:18.2 months (8.93-NA) mOS: NR ORR:84% (63.9–95.5%)	2023 (76)
First-line	NCT03050814 (QUILT-2.004/AdCEA vaccine)	II	Prospective, randomized	Chemotherapy + Anti-VEGF + Anti-PD-L1 + AdCEA vaccine	**Experimental arm:**20% **Control arm:** 70%	**Experimental arm:**20% **Control arm:**0%	NR	**Experimental arm:**10 **Control arm:** 10	**Experimental arm:** Avelumab + AdCEA vaccine + mFOLFOX6 + Bevacizumab **Control arm:** FOLFOX plus Bevacizumab	**Experimental arm:** mPFS:10.1 months (3.6–16.1) mOS: NR ORR:50% (NR) **Control arm:** mPFS:8.8 months (3.3–17.0) mOS: NR ORR:50% (NR)	2022 (77)
First-line maintenance	NCT02291289 (MODUL)	II	Prospective, multicenter, randomized, double-blind	Chemotherapy+Anti-VEGF+Anti-PD-L1	**Experimental arm:**60.5% **Control arm:** 65.3%	0%	**Experimental arm:**78.8% **Control arm:** 75.0%	**Experimental arm:** 297 **Control arm:**148	**Experimental arm:** Fluoropyrimidine + Bevacizumab+ Atezolizumab **Control arm:** Fluoropyrimidine + Bevacizumab	**Experimental arm:** mPFS:7.1 months (6.1-8.3) mOS: NE (17.9-NE) ORR:13.8% (10.1-18.3%) **Control arm:** mPFS 7.4 months (5.9-9.1) mOS: NE (18.8-NE) ORR 12.2% (7.4-18.5%)	2022 (78)
First-line	NCT02671435	I/II; reported data from first-line MSS-CRC dose-exploration cohort	Prospective, multicenter	Chemotherapy+Anti-VEGF+Anti-PD-L1+Anti-NKG2A	NR	NR	NR	18 enrolled; 17 efficacy-evaluable	mFOLFOX6+Bevacizumab+Durvalumab+Monalizumab	mPFS: NR mOS: NR ORR:41.2% (NR)	2020 (55)

NR, not reported; NE, not estimable; NA, not applicable; mPFS, median progression-free survival; mOS, median overall survival; ORR, overall response rate; MEK, mitogen-activated extracellular signal-regulated kinase; HDAC, histone deacetylase; RP2D, recommended phase 2 dose; FMT, fecal microbiota transplantation; FOLFOX, folinic acid+5-fluorouracil+oxaliplatin regimen; FOLFOXIRI, folinic acid+5-fluorouracil+oxaliplatin+irinotecan regimen; CAPOX, capecitabine+oxaliplatin regimen; SOC, standard of care; NKG2A, natural killer group 2 member A; AdCEA, vaccine adenovirus-based carcinoembryonic antigen vaccine; CEA, carcinoembryonic antigen; MSS, microsatellite stable; MSI, microsatellite instability.

*Bold values represent primary efficacy endpoints of the corresponding clinical trials, including ORR, mPFS and mOS.

#### 2.2.1 ICI + VEGF/VEGFR inhibition + chemotherapy

Although chemotherapy combined with anti-VEGF therapy remains a standard first-line treatment for pMMR/MSS metastatic colorectal cancer, its clinical benefit is not durable in a substantial proportion of patients. These limitations have prompted investigation of whether the addition of immune checkpoint blockade could further enhance the depth and durability of response (35).

The AtezoTRIBE study showed that the addition of atezolizumab to first-line FOLFOXIRI plus bevacizumab might improve progression-free survival in patients with metastatic colorectal cancer (36). In the initial analysis of the pMMR subgroup, patients receiving atezolizumab in addition to FOLFOXIRI plus bevacizumab had longer median PFS than those receiving FOLFOXIRI plus bevacizumab alone (mPFS, 12.9 vs. 11.4 months; HR, 0.78; 80% CI, 0.62–0.97; P = 0.071; mOS, 30.8 vs. 29.2 months; HR, 0.80; 80% CI, 0.63–1.02; P = 0.117) (36), indicating only modest improvements in both PFS and OS. Exploratory biomarker analyses further identified tumor mutational burden (TMB) and immune-cell score as potentially promising biomarkers (36). These findings suggest that patients with high TMB, a high immune-cell score, or both may represent a subgroup more likely to benefit from the addition of immune checkpoint blockade to first-line therapy and may therefore warrant enrichment in future clinical trials (36).

In this respect, the NIVACOR study showed that adding nivolumab to FOLFOXIRI plus bevacizumab produced a high response rate as first-line treatment in patients with RAS/BRAF-mutated mCRC, irrespective of MMR status (37). In the pMMR/MSS cohorts, the ORRs were 77.8%(95% CI, 65.5-87.3%) and DCRs were 96.8%(95% CI, 89.0-99.6%) (37). Meanwhile, some findings are broadly consistent with previous observations from AtezoTRIBE, in which greater benefit from the addition of atezolizumab to FOLFOXIRI plus bevacizumab was observed in patients with high TMB or a high Immunoscore (37). However, larger randomized studies with prospective biomarker validation are still required to confirm these findings and better define the role of this combination in the treatment landscape of mCRC.

The phase II CheckMate 9X8 trial showed that adding nivolumab to standard-of-care chemotherapy plus bevacizumab did not result in a statistically significant improvement in median PFS compared with SOC alone in the first-line treatment of mCRC (35). However, a higher PFS rate was observed beyond 12 months in the nivolumab-containing arm, together with a higher response rate and more durable responses (35). The safety profile of nivolumab plus SOC was consistent with the known toxicities of the individual chemotherapy and immunotherapy components, with no new safety signals identified (35). Exploratory subgroup analyses further suggested that patients with the CMS3 subtype may derive greater benefit from nivolumab plus SOC, indicating that molecular subtype may have potential value for patient stratification in future studies (35).

The BACCI trial met its prespecified primary endpoint, with the addition of atezolizumab to capecitabine plus bevacizumab resulting in a modest improvement in PFS (38). Median PFS was 5.3 months with atezolizumab versus 3.3 months without atezolizumab (HR, 0.66; 95%CI, 0.44–0.99) (39). No new or unexpected safety signals were observed (38). Notably, a *post hoc* analysis showed a higher ORR among patients without liver metastases than among those with liver metastases in the atezolizumab group (23.1% [3/13] vs 5.8% [4/69], P = 0.04) (38). However, the treatment-by-liver-metastasis interaction for PFS was not statistically significant (P for interaction = 0.68) (38).

#### 2.2.2 ICI + VEGF/VEGFR inhibition + intensified immune checkpoint blockade

Combining VEGF/VEGFR-targeted treatment with blockade of both PD-1 and CTLA-4 is another approach under investigation, with or without chemotherapy. In the phase I RIN trial, patients with refractory MSS mCRC received regorafenib, nivolumab and ipilimumab. Among the 29 patients treated at the recommended phase II dose, the ORR was 27.6%(95% CI, no reported) (8/29), median PFS was 4.0 months (95% CI, 3.0–9.0), and median OS was 20.0 months (95% CI, 9.0 months to not reached) (40). All eight objective responses occurred in patients without liver metastases (8/22 vs 0/7), although these small subgroups do not establish liver-metastasis status as a predictor of benefit (40).

The phase II AtezoTRIBE study demonstrated that the addition of atezolizumab to first-line FOLFOXIRI plus bevacizumab could improve clinical outcomes in patients with metastatic colorectal cancer, though the survival benefit remained modest in the pMMR/MSS subgroup (41). To further potentiate immune checkpoint blockade in this population, the multicenter, single-arm phase II SYLT-026 study investigated the first-line efficacy of cadonilimab, a bispecific PD-1/CTLA-4 antibody, in combination with FOLFOXIRI and bevacizumab (42). The latest updated data showed that all 20 enrolled patients achieved partial responses, yielding an objective response rate of 100%(95% CI, no reported) (42). After a median follow-up of 24.4 months, the median progression-free survival reached 17.2 months, while overall survival data remained immature (42, 43). In terms of safety, most adverse events were grade 1–2, and immune-related adverse events occurred in 30% of patients. Collectively, the combination of cadonilimab with FOLFOXIRI and bevacizumab exhibits promising antitumor activity and a manageable safety profile as first-line therapy for pMMR/MSS metastatic colorectal cancer, which warrants further validation in large-scale randomized phase III trials (42).

#### 2.2.3 ICI + VEGF/VEGFR inhibition + epigenetic modulation

HDAC inhibitors can increase the activity of NK cells and CD8^+^ T cells while reducing the abundance and suppressive function of Tregs (44). Histone deacetylase inhibition may also improve antigen presentation and alter immune-cell infiltration, providing a rationale for combining HDAC inhibition with checkpoint blockade in pMMR/MSS tumors (45–47).

The randomized phase II CAPability-01 trial evaluated sintilimab plus the histone deacetylase inhibitor chidamide with or without bevacizumab in patients with chemotherapy-refractory MSS/pMMR advanced colorectal cancer (48). The study met its primary endpoint, and the addition of bevacizumab significantly improved clinical outcomes, with an 18-week PFS rate of 64.0% versus 21.7%, an ORR of 44.0% versus 13.0%(95% CI, 24.4-65.1% vs 2.8-33.6%), and a median PFS of 7.3 versus 1.5 months compared with sintilimab plus chidamide alone (48). Notably, this benefit was also observed in patients with liver metastases (48). RNA sequencing analysis further suggested increased CD8^+^ T-cell infiltration and upregulation of pathways involved in antigen presentation and antitumor immune responses in the triplet arm, suggesting that simultaneous PD-1 blockade, HDAC inhibition, and VEGF targeting may remodel the immunosuppressive tumor microenvironment toward a more immunologically active state and enhance the efficacy of immunotherapy in refractory MSS/pMMR mCRC (48).

#### 2.2.4 ICI + VEGF/VEGFR inhibition + MAPK pathway inhibition

MEK inhibition may increase MHC-I expression, reduce immunosuppressive mediators such as VEGF, IL-1 and IL-8, and alter the balance of myeloid and T-cell populations in the tumor microenvironment (49, 50).In a single-institution phase II study, pembrolizumab plus binimetinib and bevacizumab achieved an ORR of 12%(95% CI,4.5–24.3%) in heavily pretreated BRAF wild-type MSS mCRC (51). Although the study did not meet its prespecified primary endpoint compared with the 5% historical control threshold, the regimen achieved a DCR of 70%(95% CI, 55.4-82.1%) and a median PFS of 5.9 months, with the longest partial response lasting 20.1 months, indicating clinically meaningful disease control in a subset of patients (51). Notably, greater clinical activity was observed in patients without liver metastases, who showed a trend toward higher response rates and significantly longer OS than those with liver metastases (51). These findings suggest that this combination may have greater potential in heavily pretreated MSS mCRC patients without liver metastases, although further validation is required (51).

#### 2.2.5 ICI + VEGF/VEGFR inhibition + adenosine-pathway blockade

The phase Ib/II COLUMBIA-1 study evaluated the addition of durvalumab and the anti-CD73 antibody oleclumab to first-line FOLFOX plus bevacizumab in patients with untreated MSS mCRC (52). The experimental regimen produced a numerically higher ORR than FOLFOX plus bevacizumab alone (61.5% vs 46.2%(95% CI, 40.6-79.8% vs 26.6-66.6%)), whereas DCRs were similar between the two groups (84.6% vs. 88.5%(95% CI, 65.1–95.6% vs 69.8–97.6%) (52). However, the improvement in response rate did not translate into longer PFS or OS (52). Exploratory biomarker analyses suggested that patients with high tumor CD73 expression had numerically higher response rates with durvalumab plus oleclumab and appeared to have longer PFS than patients with low CD73 expression, although these findings were limited by the small sample size and lack of stratification by baseline CD73 expression (52). Overall, the efficacy results did not support further development of the durvalumab/oleclumab/FOLFOX and bevacizumab combination in MSS mCRC (52).

#### 2.2.6 ICI + VEGF/VEGFR inhibition + NKG2A blockade

NKG2A is an inhibitory receptor expressed on NK cells and a subset of CD8^+^ T cells, and blockade of the NKG2A–HLA-E axis with monalizumab may complement PD-L1 blockade by relieving suppression of both innate and adaptive cytotoxic responses (53, 54). In a phase I/II study, a dose-exploration cohort evaluated durvalumab plus monalizumab in combination with mFOLFOX6 and bevacizumab as first-line treatment for advanced/metastatic MSS CRC (55). Among 17 response-evaluable patients, the confirmed ORR was 41.2% (7/17)(95% CI, no reported), with an additional two patients achieving unconfirmed partial responses, while 47.1% achieved stable disease (55). The regimen showed preliminary antitumor activity with a manageable safety profile, although these findings require confirmation in larger clinical trials (55).

#### 2.2.7 ICI + VEGF/VEGFR inhibition + innate immune stimulation

Imprime PGG is designed to activate dendritic cells, monocytes/macrophages, and NK cells and to promote antigen-specific CD8^+^ T-cell priming (56, 57). In the randomized phase Ib/II MORPHEUS-CRC study, patients with refractory MSS mCRC received atezolizumab plus Imprime PGG and bevacizumab or regorafenib (58). No objective responses were observed in either arm (58). The DCR was 13% versus 23%, median PFS was 1.5 versus 2.8 months, and median OS was 5.7 versus 10.2 months, respectively (58). Thus, despite its biological rationale and acceptable tolerability, the Imprime-containing regimen did not demonstrate a meaningful efficacy signal in this population (58).

#### 2.2.8 ICI + VEGF/VEGFR inhibition + microbiome modulation

RENMIN-215 was the first prospective study to evaluate tislelizumab, an anti-PD-1 antibody, plus the VEGFR inhibitor fruquintinib and fecal microbiota transplantation (FMT) as third-line or later therapy for patients with MSS mCRC (59). In the updated analysis, the regimen maintained an ORR of 20.0%(95% CI, 5.7–43.7%) and a median OS of 13.7 months. Compared with a real-world cohort receiving later-line ICI-based therapy, propensity score–matched analysis showed a significantly longer OS with the RENMIN-215 regimen (21.2 vs. 15.8 months; HR, 0.26; 95% CI, 0.07–0.95; P = 0.042), although the PFS difference was not statistically significant (59). The combination was generally well tolerated, with manageable treatment-related adverse events and no new safety concerns; most adverse events were low grade (59). Overall, these updated findings support sustained antitumor activity of tislelizumab plus fruquintinib and FMT in heavily pretreated MSS mCRC and warrant further evaluation in controlled clinical trials (59).

#### 2.2.9 Safety

Multi-agent regimens generally produced a higher toxicity burden than dual-agent therapy, particularly when chemotherapy was incorporated. Grade≥3 adverse events were reported in 65.8% of patients in NIVACOR, 75% in CheckMate 9X8, 52% as grade 3–4 TRAEs in POCHI, and 69.1% in ASTRUM-015, with diarrhea, fatigue, neutropenia, anemia, and nausea among the most common toxicities. Additional targeted or immune-modulatory agents also introduced mechanism-specific toxicities, including acneiform rash with MEK inhibition, thrombocytopenia with HDAC inhibition, and infusion-related reactions with Imprime PGG. The safety of multi-agent therapy is influenced by toxicities associated with the additional treatment components. Therefore, clinical treatment selection should balance potential efficacy gains against cumulative toxicity (**Table 4**).

**Table 4:** Safety profiles of multi-agent regimens based on immune checkpoint inhibitors and VEGF/VEGFR-targeted therapy.

Targets	Clinical trial	Regimen	Safety population	Grade≥3 AEs, n (%)	common AEs
Chemotherapy + anti-VEGF + PD-1	NCT04072198 (37) (NIVACOR)	FOLFOXIRI + Bevacizumab + Nivolumab	73	48 (65.8%)	Diarrhea, fatigue, neutropenia
	NCT05839470 (42) (SYLT-026)	Cadonilimab + FOLFOXIRI/FOLFIRINOX + Bevacizumab	20	NR	Stomatitis, proteinuria, diarrhea
	NCT06206096 (74)	Sintilimab + Bevacizumab + CAPOX	33	NR	Anemia, vomiting/nausea, neutropenia
	NCT03414983 (35) (CheckMate 9X8)	Nivolumab + mFOLFOX6 + Bevacizumab	127	75%	fatigue, nausea, Peripheral neuropathy
	NCT04547166 (75) (ASTRUM-015)	Serplulimab + HLX04 + XELOX	55	39 (70.9%)	Anemia, platelet count decreased, neutrophil count decreased
	Gao et al., 2024 (73)	Chemotherapy + Bevacizumab + anti-PD-1	29	NR	Anemia, leukopenia, palmar-plantar erythrodysesthesia
	NCT03396926 (71)	Pembrolizumab + Capecitabine + Bevacizumab	44	13 (30%)	Palmar-plantar erythrodysesthesia
Chemotherapy + anti-VEGF + PD-L1	NCT03721653 (36) (AtezoTRIBE)	FOLFOXIRI + Bevacizumab + Atezolizumab	145	95 (66%)	neutropenia, diarrhea, febrile neutropenia
	NCT04068610 (52) (COLUMBIA-1)	FOLFOX + Bevacizumab + Durvalumab + Oleclumab	26	17 (65.4%)	diarrhea, nausea, constipation
	NCT03050814 (77) (QUILT-2.004)	Avelumab + AdCEA vaccine + mFOLFOX6 + Bevacizumab	10	NR	Lymphocyte count decreased, Fatigue, Infusion-related reaction
	NCT02671435 (55)	mFOLFOX6 + Bevacizumab + Durvalumab + Monalizumab	18	NR	Fatigue, nausea, peripheral neuropathy
	NCT04194359 (76) (BBCAPX)	Sintilimab + Bevacizumab + CAPOX	25	NR	Anemia, Neutropenia, Nausea
	NCT02291289 (78) (MODUL)	Fluoropyrimidine + Bevacizumab + Atezolizumab	297	NR	hypertension, diarrhea, palmar-plantar erythrodysesthesia
	NCT02873195 (38) (BACCI)	Capecitabine + Bevacizumab + Atezolizumab	82	53(61.6%)	Hypertension, diarrhea, palmar-plantar erythrodysesthesia
Chemotherapy + anti-VEGF + dual/other immune modulation	NCT05627635 (72) (FOLFOX-3B)	FOLFOX + Bevacizumab + Botensilimab + Balstilimab	14	1(7.1%)	AST/ALT elevation, hyperthyroidism
TKI/anti-VEGF + PD-(L)1 + another intervention	NCT04362839 (40) (RIN)	Regorafenib + Ipilimumab + Nivolumab	39 total; 29 at RP2D	NR	Maculopapular rash, AST elevation, ALT elevation
	NCT03555149 (58) (MORPHEUS-CRC)	Atezolizumab + Imprime PGG + Bevacizumab	15	13%	Infusion-related reaction, diarrhea, fatigue
	NCT03475004 (51)	Pembrolizumab + Binimetinib + Bevacizumab	50	32 (64%)	Acneiform rash, diarrhea, nausea
	NCT04724239 (48) (CAPability-01)	Chidamide + Sintilimab + Bevacizumab	25	13 (52%)	Thrombocytopenia, proteinuria, neutropenia
	ChiCTR2100046768 (59) (RENMIN-215)	Fruquintinib + Tislelizumab + FMT	20	7 (35.0%)	Decreased appetite, albuminuria, fecal occult blood positive

NR, not reported; RP2D, Recommended Phase 2 Dose; AST, Aspartate Aminotransferase; ALT, Alanine Aminotransferase; FOLFOXF, olinic acid+5-Fluorouracil+Oxaliplatin; CAPOX(XELOX), Capecitabine + Oxaliplatin; mFOLFOX6, Modified FOLFOX6; FOLFOXIRI, Folinic acid+5-Fluorouracil+Oxaliplatin+Irinotecan; FMT, fecal microbiota transplantation.

## 3 Discussion

Approximately 95 % of patients with advanced colorectal cancer harbor pMMR/MSS tumors, these immune-cold malignancies derive minimal clinical benefit from single-agent immune checkpoint inhibitors (8). Although multi-target tyrosine kinase inhibitors are applied in later-line settings for advanced colorectal cancer to delay tumor progression, their objective response rate is merely 1% (8, 15). Against this backdrop, combining immune checkpoint inhibitors with VEGF/VEGFR-targeted therapy has emerged as a promising research direction. Several clinical cohorts have observed encouraging tumor responses with such combinations; nevertheless, phase III trial outcomes remain inconsistent, and clinical benefits are limited within unselected overall patient populations (29, 30). Given this situation, the present review addresses two core scientific questions: what are the real-world clinical efficacies of dual-agent and multi-agent combination regimens, and which clinical and molecular features identify patients more likely to benefit from these combinatorial therapies. A review of available evidence demonstrates that dual-agent combinations showed numerically higher objective response rates compared with standard third-line therapies for pMMR/MSS patients. Among multi-agent strategies, different regimens vary in their capacity to remodel the tumor immune microenvironment, which contributes to substantial heterogeneity in clinical outcomes. Multiple factors including intrinsic tumor molecular features, metastatic patterns and prior treatment history jointly shape therapeutic responses. Accordingly, developing robust predictive biomarkers and precisely identifying potential beneficiaries for distinct combination regimens represents a core priority for future research, which may help address the unsatisfactory immunotherapy outcomes observed in pMMR/MSS colorectal cancer.

Early phase II single-arm trials such as REGONIVO reported an objective response rate of 36%(95% CI, 18–57.5%) with regorafenib plus nivolumab, generating high expectations for this treatment paradigm (19). Subsequent phase II studies including regorafenib combined with sintilimab and fruquintinib plus sintilimab also demonstrated promising response and survival signals (22). However, these positive observations from small non-randomized cohorts have not been consistently reproduced in large-scale phase III trials. In the LEAP 017 trial, lenvatinib plus pembrolizumab yielded numerically higher objective response rate and duration of response versus standard of care later line treatments but failed to meet the pre specified overall survival endpoint (29). By contrast, the STELLAR-303 trial reported statistically significant overall survival improvement with zanzalintinib plus atezolizumab versus regorafenib, marking the first positive phase III overall-survival result in this refractory pMMR/MSS patient population identified in this review (30). One proposed explanation relates to differing kinase inhibition profiles: besides VEGFR, zanzalintinib inhibits MET and TAM family kinases AXL and MER, which may further counteract myeloid cell driven immune suppression (31). However, because these findings were derived from separate trials involving different agents, patient populations, and study designs, cross-trial comparisons cannot establish that the broader kinase-inhibition profile of zanzalintinib directly accounted for the positive outcome of STELLAR-303. Further prospective head-to-head studies are therefore needed to determine whether differences in kinase-inhibition profiles translate into clinically meaningful differences in efficacy. Notably, even with the same TKI regorafenib, objective response rates range from 0 %(95% CI, no reported) in the REGOMUNE trial (regorafenib + avelumab) (25) to 7 %-21.4 % across other regorafenib-plus-PD-1 studies (14, 24). Variables such as drug dosing and baseline tumor-microenvironment features including M2-macrophage and Treg abundance likely shape treatment results. Single-arm phase II data tend to overestimate real-world therapeutic activity and should therefore be interpreted prudently.

Exploratory subgroup analyses from multiple independent trials identify liver-metastasis status as a repeatedly observed candidate prognostic factor for response to ICI-anti-angiogenic combinations. Across studies evaluating regorafenib plus nivolumab, atezolizumab plus bevacizumab and anlotinib plus sintilimab, patients without liver metastases consistently achieve higher objective response rates; in several cohorts, all confirmed objective tumor responses occur among patients free of hepatic disease. Mechanistic work indicates that liver metastases can establish a systemically immunosuppressive state by triggering macrophage-mediated elimination of effector T cells, which partly accounts for inferior immunotherapy responses in patients with hepatic involvement (26). RAS/RAF molecular status also shows potential predictive value: patients with wild-type RAS tend to derive greater benefit compared with RAS-mutant counterparts, though tumor responses can still occur in RAS-mutant cases and mutation status cannot serve as an absolute exclusion criterion (21, 22, 60). It must be emphasized that these observations stem largely from post-hoc exploratory analyses rather than pre-specified trial stratification. Of note, benefit was maintained even among patients with liver metastases in the CAPability-01 triple-arm study (48), implying that multi-agent intervention might partially reverse liver-metastasis-related immune resistance. Accordingly, liver-metastasis status should not be used as an absolute contraindication for these regimens, and prospective validation is required.

Given the modest overall response depth and durability observed with dual-agent ICI-anti-angiogenic regimens, researchers have moved toward multi-agent combinatorial strategies, the core idea is to add agents with non-overlapping mechanisms aiming at multiple nodes of the immune-suppressive circuitry to convert immune-cold tumors, rather than only exerting direct cytotoxicity. A broad spectrum of therapeutic partners has been explored, including chemotherapy agents, enhanced immune-checkpoint blockers, epigenetic modulators, MAPK-pathway inhibitors and gut-microbiota-targeted interventions.

Among these triple-agent approaches, chemotherapy combined with bevacizumab and ICIs represents the most-investigated first-line strategy. AtezoTRIBE and CheckMate 9X8 show only limited survival increments when ICIs are added to standard chemo-anti-angiogenic backbone for unselected pMMR/MSS patients. Nevertheless, subgroups defined by high tumor mutational burden, high Immunoscore and CMS3 subtype exhibit more favorable therapeutic signals, supporting biomarker-enriched trial designs to reduce signal dilution caused by patient heterogeneity (35–37, 61). The phase II SYLT-026 study applied PD-1/CTLA-4 bispecific cadonilimab plus FOLFOXIRI-bevacizumab and reported encouraging efficacy, though validation in larger phase III cohorts remains needed (42). The randomized CAPability-01 trial demonstrated substantial clinical gains by adding bevacizumab to sintilimab-chidamide, with efficacy preserved in patients with liver metastases (48).

Other multi-agent concepts have delivered mixed results. The RENMIN-215 trial suggested survival benefit from fecal microbiota transplantation combined with fruquintinib-tislelizumab in heavily pre-treated patients, yet conclusions are limited by its single-arm design (59). Meanwhile, biologically attractive strategies assessed in COLUMBIA-1 and MORPHEUS-CRC failed to translate preclinical rationale into meaningful clinical survival endpoints (52, 58). These examples highlight that promising mechanistic background does not guarantee clinical success. In addition, multi-component regimens carry risks of additive grade ≥ 3 adverse events. Balancing antitumor activity and tolerability deserves special attention, especially for elderly, heavily pre-treated subjects with poor ECOG performance status.

A major bottleneck in this field is the absence of prospectively validated biomarkers for selecting pMMR/MSS colorectal cancer patients most likely to respond to ICI VEGF/VEGFR targeted combinations. Rather than relying on a single biomarker, current evidence suggests that treatment sensitivity may reflect the combined effects of tumor immunogenicity, immune-cell infiltration, systemic immune status, and dynamic treatment-induced changes. Tumor mutational burden (TMB) and Immunoscore represent two of the most frequently investigated indicators of baseline tumor immunogenicity. Exploratory analyses from trials such as AtezoTRIBE and CheckMate 9X8 suggest that patients with higher TMB, greater immune-cell infiltration, or selected immune-enriched molecular subtypes may derive greater benefit from the addition of ICIs (35–37, 61). In parallel, the composition of the tumor microenvironment may also be informative. In REGOMUNE, high baseline tumor-associated macrophage infiltration was significantly associated with shorter PFS and OS (25), whereas a biomarker analysis of regorafenib plus nivolumab found that lower pretreatment Treg infiltration correlated with prolonged PFS (20). However, these associations have not been consistently reproduced across studies, highlighting the complexity of using individual immune-cell populations as standalone predictive biomarkers.

Dynamic biomarkers may provide additional information that cannot be captured by baseline tumor characteristics alone. In the RIN trial, ctDNA dynamics were assessed in 23 patients, and a decrease of more than 50% in ctDNA was associated with clinical benefit, supporting the potential value of ctDNA as an early response biomarker (40). The investigators further suggested that failure to achieve a ctDNA decrease by 6 weeks may help identify patients unlikely to benefit from continued treatment.

Similarly, gut-microbiome composition has emerged as a potential determinant of immunotherapy responsiveness, and the clinical activity observed with microbiota-modulating strategies such as RENMIN-215 provides preliminary support for further investigation of microbial signatures in this setting (59). However, these biomarkers have generally been evaluated in small exploratory cohorts, using heterogeneous assays, thresholds, and sampling schedules, and few have undergone independent or prospective validation. Consequently, no biomarker or biomarker panel can currently identify with sufficient reliability which pMMR/MSS patients will benefit from ICI–VEGF/VEGFR combinations. This represents a major translational gap between the biological rationale of tumor-microenvironment remodeling and its clinical application. Future trials should therefore integrate prospective, longitudinal biomarker assessment—including tumor tissue, peripheral blood, ctDNA, cytokine profiles, and microbiome features—to determine not only which patients are immunologically permissive at baseline, but also whether treatment induces a measurable shift toward a more immune-responsive tumor state.

From a safety perspective, toxicities of dual-agent combinations are largely influenced by the type of VEGF/VEGFR-targeted agent used. TKI-based regimens are commonly associated with hypertension, proteinuria, hand-foot syndrome, diarrhea, and fatigue, reflecting the broader inhibition of VEGFR and, in some cases, additional kinase targets. Immune-related adverse events, including rash, hypothyroidism, and hepatic dysfunction, are also observed. In contrast, combinations of ICIs with the anti-VEGF antibody bevacizumab are more commonly associated with fatigue, hypertension, and abdominal pain. Hypertension is a well-recognized class effect of VEGF inhibition and is thought to result primarily from reduced VEGF-dependent endothelial nitric oxide and prostacyclin production, leading to impaired vasodilation and increased vascular resistance. Fatigue is likely multifactorial, reflecting systemic effects of anticancer treatment, immune activation, and the underlying disease, whereas abdominal pain is less mechanism-specific and may relate to gastrointestinal effects of VEGF inhibition, underlying colorectal disease, or concomitant treatment. Therefore, these adverse events should not all be attributed solely to bevacizumab itself.

Triple-agent regimens incorporating chemotherapy or multiple immune modulators carry further elevated risks of myelosuppression, gastrointestinal toxicity and immune-related adverse events, requiring close clinical monitoring. At present, ICI-VEGF/VEGFR-targeted combinations are not established standard-of-care for pMMR/MSS metastatic colorectal cancer and remain largely investigational. Universal off-label application across all MSS patients is not recommended, and eligible patients should be prioritized for clinical-trial participation.

Beyond efficacy and safety considerations, multi-agent regimens raise substantial economic concerns. Combinations stacking multiple novel agents are associated with high treatment costs, limiting real-world accessibility even for patients who might derive clinical benefit. Cost-effectiveness analyses will therefore become essential before broad clinical adoption of these triple-agent strategies.

## 4 Limitations

This review has several limitations. First, a substantial proportion of included studies are phase I/II single-arm open-label trials lacking parallel control groups, which may introduce selection bias, wide confidence intervals and are prone to random fluctuation. Second, included trials vary widely with respect to agent choice, dosing, treatment lines and baseline patient characteristics, prohibiting direct cross-study comparison of regimen performance. Third, many subgroup observations derive from post-hoc, non-pre-specified analyses. Such analyses carry risks of multiplicity-driven inflated type-I error alongside limited sample sizes; accordingly, these findings require prospective validation in dedicated trials. Fourth, long-term follow-up remains immature in several studies, and head-to-head comparisons between different ICI–VEGF/VEGFR strategies are lacking. Fifth, this review relies exclusively on fully published peer-reported data and conference abstracts; unpublished negative or neutral study results were not retrieved, which may lead to publication bias.

## 5 Conclusion and perspectives

Available clinical evidence suggests that dual-agent regimens combining ICIs with VEGF/VEGFR-targeted therapy can achieve tumor response and survival benefits in subsets of patients with advanced pMMR/MSS colorectal cancer. However, treatment efficacy varies substantially across clinical trials and phase III outcomes remain inconsistent. Clinical features such as absence of liver metastases and wild-type RAS status correlate with a higher likelihood of treatment response, yet these observations are mostly derived from exploratory subgroup analyses and should be further validated in larger prospective biomarker-enriched trials.

Furthermore, treatment-line status constitutes another repeatedly noted potential confounding variable. Heavily pretreated late-line patients often harbor greater tumor-intrinsic resistance and cumulative immune impairment from prior chemotherapy, which may diminish treatment responses when compared with patients receiving the same combinations in earlier-line settings.

To improve therapeutic outcomes, multiple triple-agent strategies have been investigated, including combinations with chemotherapy, dual immune-checkpoint blockade, epigenetic modulation, and gut-microbiota intervention. Several of these approaches have yielded encouraging antitumor activity, though most remain at phase II development. In addition, additive toxicities associated with multi-agent combinations warrant close attention. To date, no prospectively validated biomarkers are available for precise identification of potential responders, and such combination regimens have not yet become standard-of-care for pMMR/MSS metastatic colorectal cancer.

Future research should prioritize large-scale randomized phase III trials to verify promising strategies including the chidamide-based triple regimen and cadonilimab combined with chemotherapy plus anti-angiogenic agents. It is essential to develop and prospectively validate predictive biomarkers and implement biomarker-enriched trial designs to select patients most likely to derive clinical benefit. Novel multi-target kinase inhibitors, bispecific antibodies and gut-microbiota-modulating interventions also merit further exploration. Collectively, these multi-pronged efforts may help transform immune-cold pMMR/MSS tumors into immune-sensitive phenotypes and broaden therapeutic choices for patients with advanced colorectal cancer.

## Glossary

AE: adverse event
AdCEA vaccine: adenovirus-based carcinoembryonic antigen vaccine
ALT: Alanine Aminotransferase
AMP: adenosine monophosphate
AST: Aspartate Aminotransferase
CSCO: Chinese Society of Clinical Oncology
CAPOX: capecitabine + oxaliplatin
CEA: carcinoembryonic antigen
CI: confidence interval
CMS: consensus molecular subtype
CR: complete response
ctDNA: circulating tumor DNA
DCR: disease control rate
DOR: duration of response
ECOG: Eastern Cooperative Oncology Group
ESMO: European Society for Medical Oncology
FMT: fecal microbiota transplantation
FOLFOX: folinic acid + 5-fluorouracil + oxaliplatin
FOLFOXIRI: folinic acid + 5-fluorouracil + oxaliplatin + irinotecan
GSEA: gene set enrichment analysis
HDAC: histone deacetylase
HLA-E: human leukocyte antigen-E
HR: hazard ratio
ICI: immune checkpoint inhibitor
IHC: immunohistochemistry
irAE: immune-related adverse event
ITT: intention-to-treat
LAG-3: lymphocyte-activation gene-3
MAPK: mitogen-activated protein kinase
MDSC: myeloid-derived suppressor cell
NKG2A: natural killer group 2 member A
PD: progressive disease
PR: partial response
PS: performance status
RECIST: Response Evaluation Criteria in Solid Tumors
RP2D: recommended phase 2 dose
SD: stable disease
SOC: standard of care
TAM: tumor-associated macrophage
TGF-β: transforming growth factor-beta
TKI: tyrosine kinase inhibitor
TIM-3: T-cell immunoglobulin and mucin-domain-containing protein-3
TRAE: treatment-related adverse event
XELOX: capecitabine + oxaliplatin
mFOLFOX6: modified FOLFOX6
